# The multiple cellular functions of the oncoprotein Golgi phosphoprotein 3

**DOI:** 10.18632/oncotarget.3051

**Published:** 2015-01-23

**Authors:** Stefano Sechi, Anna Frappaolo, Giorgio Belloni, Gianni Colotti, Maria Grazia Giansanti

**Affiliations:** ^1^ Istituto di Biologia e Patologia Molecolari del CNR, Dipartimento di Biologia e Biotecnologie, Sapienza Università di Roma, 00185 Roma, Italy; ^2^ Dipartimento di Biologia e Biotecnologie, Sapienza Università di Roma, 00185 Roma, Italy; ^3^ Istituto di Biologia e Patologia Molecolari del CNR, Dipartimento di Scienze Biochimiche, Sapienza Università di Roma, 00185 Roma, Italy

**Keywords:** GOLPH3, cancer, Golgi

## Abstract

The highly conserved Golgi phosphoprotein 3 (GOLPH3) protein, a component of Trans-Golgi Network (TGN), has been defined as a “first-in-class Golgi oncoprotein” and characterized as a Phosphatidylinositol 4-phosphate [PI(4)P] effector at the Golgi. GOLPH3 is commonly amplified in several solid tumors. Furthermore this protein has been associated with poor prognosis in many cancers. Highly conserved from yeast to humans, GOLPH3 provides an essential function in vesicle trafficking and Golgi structure. Recent data have also implicated this oncoprotein in regulation of cytokinesis, modulation of mitochondrial mass and cellular response to DNA damage. A minute dissection of the molecular pathways that require GOLPH3 protein will be helpful to develop new therapeutic cancer strategies.

## INTRODUCTION

In recent years, compelling studies have suggested that defective endocytosis may contribute to cancer disease by defective trafficking and recycling of growth factor receptors and/or cell adhesion complexes [1]. Increasing evidence indicates that aberrant expression of vesicular trafficking components is involved in tumorigenesis [1, 2]. For example several Rab GTPases and their effectors, that function as key regulators of intracellular transport, are frequently deregulated in human cancers [3, 4]. Differently from other cytoplasmic membrane oncoproteins, GOLPH3 (also referred to as GPP34, GMx33, MIDAS and Vps74p) mostly localizes to the Trans-Golgi Network (TGN) and for this reason has been defined as a “first-in-class Golgi oncoprotein” [5]. Encoded by a gene on chromosome 5p13, a chromosomal region frequently amplified in several solid tumors, human GOLPH3 was validated as a new oncoprotein through an approach that combined integrative genomics with clinopathological and functional analysis [5]. An increasing number of research studies has involved the oncogene *GOLPH3* in several cancers including melanoma, lung cancer, breast cancer, glioma, esophageal squamous cell carcinoma, colorectal cancer, prostate cancer, renal cell carcinoma, oral tongue cancer, rhabdomyosarcoma, gastric cancer, hepatocellular carcinoma, epithelial ovarian carcinoma and pancreatic ductal adenocarcinoma (Table 1) [5–26]. Moreover high GOLPH3 levels have been associated with poor survival in many cancers, providing a prognostic biomarker of tumor progression (Table 1) [5–26]. Highly conserved from yeast to humans, GOLPH3 is essential for Golgi trafficking and structure [27–29]. This protein was initially identified during proteomic studies of the Golgi apparatus and described as a phosphorylated component of the Golgi matrix [30–32]. Subsequent data demonstrated that it functions as a Phosphatidylinositol 4-phosphate [PI(4)P] effector at the Golgi [27–29]. Importantly recent studies have gained new insight into the cellular processes requiring GOLPH3 that are not limited to Golgi homeostasis and vesicle trafficking but also comprise modulation of mitochondrial mass and lipid metabolism [33–35], regulation of cytokinesis [29], cell migration [8, 11, 12] and cell survival after DNA damage [36].

**Table 1:** Solid tumors associated with GOLPH3 up-regulation

Disease	Connection with GOLPH3 protein	References
**HCC**	GOLPH3 frequently overexpressed in HCC High GOLPH3 expression associated with poor overall survival GOLPH3 expression correlated with the NF-κB signaling in two HCC patient profiles Ectopic GOLPH3 overexpression protects against cisplatin-induced apoptosis in HCC cells	[21–23]
**RCC**	GOLPH3 frequently overexpressed in RCC GOLPH3 silencing reduces migration and invasion capacity in RCC cell lines and retards xenograft tumor growth in nude mice High GOLPH3 expression associated with poor prognosis	[16]
**EOC**	GOLPH3 overexpressed in EOC High GOLPH3 expression positively correlated with advanced clinical stage	[24, 25]
**CRC**	GOLPH3 overexpressed in CRC GOLPH3 significantly associated with overall survival of patients treated with 5-FU-based adjuvant chemotherapy	[14]
**GC**	GOLPH3 overexpression significantly related to the tumor size, histological grade, distant metastasis and TNM stage GOLPH3 expression highly correlated with Akt/mTOR signaling High expression of GOLPH3 associated with poor prognosis	[19, 20]
**ESCC**	GOLPH3 significantly overexpressed in ESCC High GOLPH3 expression correlated with the progression of ESCC High GOLPH3 expression as an independent prognostic factor of ESCC patients	[13]
**PC**	GOLPH3 frequently overexpressed in PC GOLPH3 overexpression correlated with the progression of PC from hormone sensitive phase to hormone-refractory phase	[15]
**Glioma**	GOLPH3 expression associated with tumor severity GOLPH3 overexpression involved in cell migration and invasion via the mTOR-YB1 pathway High GOLPH3 expression associated with poor prognosis	[9–12]
**OTC**	GOLPH3 frequently overexpressed in OTC High GOLPH3 expression associated with poor prognosis	[17]
**BC**	GOLPH3 overexpressed in BC cells and tissues GOLPH3 overexpression associated with enhanced AKT activity and decreased FOXO1 transcription activity GOLPH3 overexpression involved in cell migration and invasion in breast cancer cells through a mechanism that depends on PI(4)P High GOLPH3 expression correlated with poor prognosis	[7, 8]
**RMS**	Expression of GOLPH3 and GOLPH3L up-regulated in the biopsy samples Proliferation of RMS cell lines affected by depletion of GOLPH3/GOLPH3L	[18]
**PDAC**	High GOLPH3 expression associated with poor overall survival	[26]
**NSCLC**	GOLPH3 overexpression significantly related to TNM stage, lymph node status and degree of differentiation High GOLPH3 expression associated with poor prognosis	[6]

[HCC] Hepatocellular carcinoma, [RCC] Renal cell carcinoma, [EOC] epithelial ovarian carcinoma, [CRC] Colorectal cancer, [GC] Gastric cancer, [ESCC] Esophageal squamous cell carcinoma, [PC] Prostate cancer, [OTC] Oral tongue cancer, [BC] Breast cancer, [RMS] Rhabdomyosarcoma. [PDAC] Pancreatic ductal adenocarcinoma, [NSCLC] Non-small cell lung cancer, [NF-kB) Nuclear factor-κB, [TNM] Tumor-node-metastasis, [5-FU] 5-fuorouracil

### Golph3 family proteins

**Figure 1:**
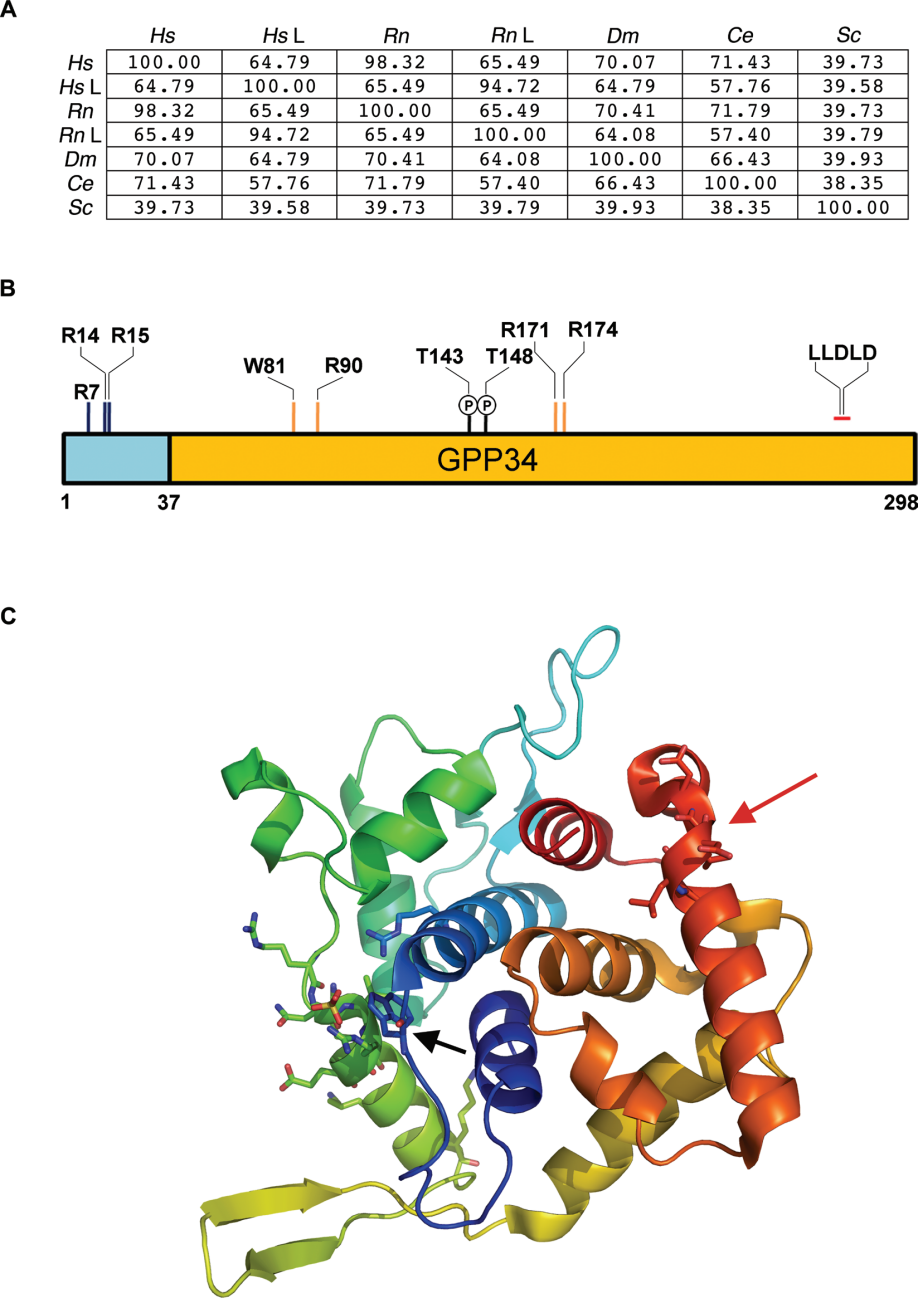
Biochemical characteristics of GOLPH3 family proteins **(A)** Percent identity matrix obtained by Clustal 2.1, comparing the GOLPH3 family proteins from several species. *Hs*, *H. sapiens*; *Rn, R.novergicus*; *Dm, D. melanogaster*; *Ce*, *C. elegans; Sc, S. cerevisiae*; *Hs* L, *H. sapiens* GOLPH3L; *Rn*L, *R. novergicus* GOLPH3L **(B)** Schematic representation of human GOLPH3 protein. (Yellow), the Gpp34 PI(4)P-binding domain;R7, R14, R15, the arginine residues required for coatomer binding; W81, R90, R171, R174, the conserved amino acid residues in the PI(4)P binding pocket.; T143, T148, the threonine residues that are phosphorylated by DNA-PK in human GOLPH3; LLDLD, the clathrin-binding motif. **(C)** Ribbon representation of the GOLPH3 protein structure (pdb: 3KN1, ref. [27]). The structure has been drawn in rainbow colors (from red: C-terminal residues, to violet: N-terminus of GOLPH3). α-helices are shown as spirals, while β-strands are indicated as arrows. The sulfate-binding pocket, indicating the putative site of PI(4)P binding, is pointed out by the black arrow. The red arrow indicates LLDLD residues of the putative clathrin-binding motif. The figure model was visualized using the Pymol software.

GOLPH3 family of Golgi proteins is highly conserved across eukaryotic kingdoms (Figure 1A) [30, 31]. Only vertebrate genomes encode two paralogs dubbed GOLPH3 (Gpp34/Gmx33α/MIDAS) and GOLPH3L (GPP34R/GMx33β) whereas lower organisms a single GOLPH3 protein [37]. The rat GOLPH3 isoforms named Gmx33α and β were identified during a proteomic analysis of an isolated Golgi fraction and characterized as detergent-insoluble proteins localized to the TGN [31]. Another proteomics study led to characterize human GOLPH3 and GOLPH3L, (also named Gpp34 and GPP34R), that were described as Golgi coat-like proteins [30]. Wu and coauthors also identified numerous predicted phosphorylation and myristoylation modifications that can influence the activity and localization of GOLPH3 [31]. Remarkably the cytoplasmic pool of GOLPH3 is less phosphorylated than the Golgi associated fraction [31]. Functional studies have recently validated putative phosphorylation sites in either yeast or mammalian cells (Figure 1B) [36, 38]. GOLPH3 proteins were characterized as PI(4)P-binding proteins through a proteomic lipid binding screen and *in vitro* lipid binding assays [27, 28]. The smallest portion of GOLPH3 that is capable of binding PI(4)P, i.e. the conserved C-terminal domain dubbed GPP34 (Figure 1B), was determined by constructing a series of fragments from *Drosophila* GOLPH3 protein, that were tested by lipid blot assay. X-ray crystal structures of the C-terminal domains of yeast and human GOLPH3 proteins have been solved, revealing a unique structure [27, 39]. Both proteins function as homo-oligomers. Indeed mutations that disrupt homo-oligomerization also affect targeting to Golgi [27, 39]. Both yeast and human GOLPH3 monomers are composed of a single globular domain that is predominantly α-helical (Figure 1C). A central four-helix bundle (α1, α2, α8, and α12) represents the core of the protein, surrounded by loops exposed to solvent and eight amphipathic helices that are roughly orthogonal to the central bundle [27, 39]. Strands β3 and β4 project as a β-hairpin away from the bulk of the helical domain of the monomer, exposing hydrophobic residues that form part of the monomer-monomer interface. Based on the crystal structure of GOLPH3 and on site-directed mutagenesis experiments, a sulfate-binding positively charged pocket on the hydrophobic face of the GPP34 domain is essential for specific binding to PI(4)P and localization of GOLPH3 proteins to the Golgi (Figure 1B, C) [28, 29]. A clathrin-binding motif (LLDLD) is located close to the C-terminus of GOLPH3 (Figure 1B, C) [29]. Multiple sequence alignment of GOLPH3 family members showed a lower degree of homology at the N-terminal region that consists of an unstructured region of 40–60 amino acids, followed by a strong homology in the GPP34 domain (Figure 1A) [37]. Both the rat and the yeast GOLPH3 proteins (Gmx33α and Vps74p respectively) are targeted to the Golgi when expressed in NRK cells as GFP fusion proteins [27, 32]. In addition human GOLPH3 can partially rescue the phenotypic defects of *vps74Δ* yeast mutants [40]. Based on these data, GOLPH3 family proteins serve an evolutionarily conserved function. Characterization of the vertebrate-specific GOLPH3 paralog GOLPH3L, in mammalian cells, revealed that this isoform is expressed at 3–10 fold lower levels than GOLPH3, with the highest concentration in secretory cells [37]. Surprisingly although GOLPH3L binds PI(4)P and localizes to the Golgi, just like GOLPH3, its action seems to be antagonistic to GOLPH3 (see below) [37].

### Golph3 and golgi homeostasis

Immunofluorescence analysis revealed that GOLPH3 localizes to Golgi structures in several organisms such as human, *Drosophila* and yeast [27–29]. Several data also indicated that GOLPH3 depends on PI(4)P, for its Golgi localization [27–29]. In the budding yeast Pik1p is the only PI 4-kinase required for PI(4)P synthesis at the Golgi [41]. Mutations affecting Pik1p impair recruitment of Vps74p to the Golgi membranes [27, 28]. Similarly the *Drosophila* PI 4-kinase IIIβ (PI4KIIIβ) Four wheel drive (Fwd) is necessary to localize GOLPH3 in spermatocytes [29]. Consistent with these data, a mutant version of GOLPH3 protein carrying amino acid substitutions in its positively charged pocket, fails to localize at the Golgi when expressed in HeLa cells or in *Drosophila* spermatocytes [28, 29].

**Figure 2:**
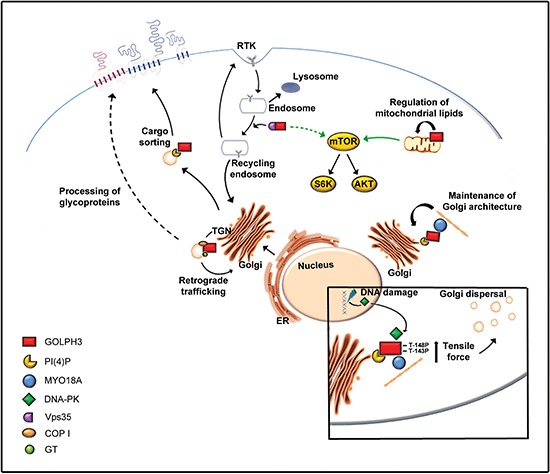
Schematic representation illustrating the main cellular functions of GOLPH3 protein GOLPH3, by binding to PI(4)P-enriched Golgi membranes and to MYO18A protein, mediates a linkage with the F-actin cytoskeleton that provides the tensile force necessary for Golgi architecture and vesicle trafficking. Golgi dispersal, in response to DNA damage, depends on the GOLPH3-MYO18A-F-actin pathway and the DNA damage protein kinase (PK) that phosphorylates GOLPH3 on T143 and T148. Several vesicle trafficking routes depend on GOLPH3 function. Human GOLPH3 also localizes to mitochondria and regulates total mitochondrial mass through mitochondrial lipid biogenesis. GOLPH3 is required for anterograde vesicle trafficking from Golgi to plasma membrane. In addition this protein associates with Vps35 a subunit of the cargo-recognition complex of the retromer which regulates receptor recycling of several transmembrane receptors including receptor tyrosine kinases (RTKs). Thus GOLPH3 might function with Vps35 and the retromer to regulate receptor recycling of key surface signals and affect downstream mTOR signaling, a pathway that is also sensitive to mitochondrial dysfunction. GOLPH3 family proteins are also essential for normal glycosylation of glycoproteins at the Golgi, a process that has been associated with several biological processes that are relevant for cancer disease including cell migration and invasion, immune recognition and signal transduction. In this context, GOLPH3 regulates docking and localization of several Golgi-resident glycosyltransferases (GT).

GOLPH3 protein is required to maintain Golgi architecture [28, 29]. In human cells, the Golgi complex is a ribbon-like system of stacks made up of flattened cisternae and occupies a perinuclear area, close to the centrosomes [42]. Knockdown of human GOLPH3 results in dramatic effects on the Golgi, changing its morphology from an “extended Golgi ribbon” to a “compact structure” at one end of the nucleus [28]. Dippold and coauthors observed similar Golgi alterations after treatment with drugs that disturb F-actin cytoskeleton [28]. Furthermore they found that GOLPH3 binds to the unconventional Myosin 18A (MYO18A), another protein required to maintain the Golgi ribbon morphology. Based on their results they proposed that human GOLPH3, by binding to PI(4)P and MYO18A, connects the Golgi to the F-actin cytoskeleton. This, in turn, supplies the tensile force required to extend the Golgi and form the ribbon-like structure (Figure 2) [28]. This model would implicate that MYO18A has active motor properties. A recent work characterized human MYO18A molecular function in the interaction with ATP/ADP, F-actin and GOLPH3 [43]. Based on this work, MYO18A contains two actin binding sites, one in the KE-rich region at the N-terminal extension and the other one in the generic motor domain which is regulated by nucleotide binding and capable to target interacting proteins to the actin cytoskeleton [43]. The PDZ module in the MYO18A N-terminal is necessary for binding to GOLPH3 and the interaction with GOLPH3 modulates the actin binding properties of the N-terminal extension. However the genome of *Saccharomyces cerevisiae* does not encode an ortholog of MYO18A and it remains to be demonstrated the interaction of Vps74p with one of the primordial myosins that are present in yeast [28, 37]. Interestingly the mammalian GOLPH3 paralog GOLPH3L also binds PI(4)P but is unable to bind to MYO18A. GOLPH3L acts as an antagonist of GOLPH3/MYO18A at the Golgi [37]. Indeed knockdown of GOLPH3L causes effects on Golgi morphology that are opposite to those observed after depletion of either GOLPH3 or MYO18A, consisting in dispersal of the Golgi membranes [37].

GOLPH3 is also required for normal Golgi structure in *Drosophila melanogaster* [29]. *Drosophila* cells, including spermatocytes, lack the ribbon-like structure [44]. Each Golgi in *Drosophila* has a paired structure consisting of two stacks held together through an actin-based mechanism [44]. Our recent work showed that wild type function of GOLPH3 is required to maintain the integrity of paired Golgi stacks in *Drosophila* cells [29].

### Golph3 and golgi reorganization induced by dna damage

DNA damage leads to the activation of several cellular well-known events that include DNA repair, cell-cycle arrest and changes of transcriptional activities [45]. Less studied are those cellular modifications, induced by DNA damage, that relate to the cytoplasmic organelles. Farber-Katz and colleagues [36] recently described a striking effect on the Golgi ribbon structure of mammalian cells, in response to the DNA damage induced by treatment with camptothecin, docorubicin or ionizing radiation. As a consequence of each DNA-damage treatment, the Golgi membranes fragment and appear dispersed throughout the cytoplasm. Remarkably the DNA-damage-induced Golgi alteration was observed in many mammalian cell lines as well as in primary cells indicating that it represents a common DNA damage response in mammalian cells [36]. The Golgi changes depend on the GOLPH3-MYO18A-F-actin pathway and the DNA damage protein kinase (DNA-PK). Specifically, GOLPH3 is phosphorylated by DNA-PK on Thr143 and Thr148 (Figure 1B), in a TQ motif. In turn phosphorylation of GOLPH3 results in enhanced interaction between GOLPH3 and MYO18A thus promoting Golgi dispersal. As a consequence of the Golgi fragmentation, anterograde vesicle trafficking from the Golgi to the plasma membrane is disrupted [36].

The effect of DNA damage on the Golgi has been largely neglected in past studies as it was thought as a consequence of DNA-damage apoptosis [46, 47]. However the study of Farber-Katz and colleagues [36] clearly demonstrated that Golgi reorganization after DNA damage is unrelated to apoptosis, rather the DNA-PK-GOLPH3-MYO18A pathway increases cell survival after DNA damage.

### Golph3 and vesicle trafficking

**Figure 3:**
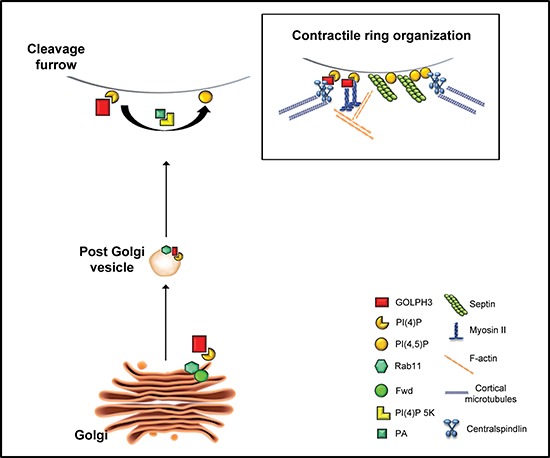
A possible model illustrating GOLPH3 function during cytokinesis Wild type function of the PI4KIIIβ (named Fwd in *Drosophila*) is required for PI(4)P synthesis at the Golgi. Recruitment of GOLPH3 to the Golgi depends on PI(4)P. Fwd protein binds Rab11 and recruits this protein on Golgi membranes. Wild type function of GOLPH3 is required to recruit PI(4)P-and Rab11-containing secretory organelles to the cleavage furrow. PI(4)P is the immediate precursor of PI(4,5)P2 that is generated in the cleavage furrow by PI(4)P 5-kinase. Binding to phosphatidic acid (PA), GOLPH3 might also contribute to PI(4)P 5-kinase activation and plasma membrane remodeling. PI(4)P-GOLPH3 and PI(4,5)P2 mediate the interaction of centralspindlin, septins and actomyosin with the plasma membrane at the cleavage site, playing a crucial role in contractile ring formation.

Although GOLPH3 is primarily enriched in the TGN, its association with the Golgi is highly dynamic [31]. Similar to other Golgi proteins, GOLPH3 depends on GTP for its localization to Golgi membranes and can be sequestered to vesicles through the use of non-hydrolyzable analogs [31]. GFP-tagged GOLPH3 was visualized on tubule vesicular structures exiting from the Golgi, on endosomes and on the plasma membrane (PM), suggesting that GOLPH3 may be involved in several vesicle trafficking steps [31]. Indeed, knockdown of human GOLPH3 by siRNA causes defects in anterograde trafficking from Golgi to plasma membrane [28]. The role of GOLPH3 proteins in vesicle trafficking pathways was further demonstrated by work in *Saccharomyces cerevisiae*. Indeed *VPS74* was discovered in a screen designed to identify synthetic lethality with *YPT6* deletion [48] and in a screen for dosage suppressors of the lethal phenotype caused by *SFT1* deletion [38]. Ypt6p, the yeast ortholog of human Rab6, regulates intra-Golgi and endosome-to-Golgi trafficking [48], whereas Sft1p is a Golgi SNARE (soluble N-ethylmaleimide-sensitive factor attachment protein receptor) required for retrograde traffic within the Golgi [49]. In addition deletion of *VPS74* disrupts transport of vacuolar proteases indicating a role for Vps74p in facilitating cargo sorting from Golgi [50]. The involvement of GOLPH3 in endocytic and secretory vesicle trafficking was also shown by our work in *Drosophila* (Figure 2 and Figure 3) [29]. In addition both human and *Drosophila* GOLPH3 proteins were shown to interact with Vps35, a component of the retromer, an evolutionary conserved endosomal complex [5, 29]. Retromer, which comprises the cargo-selective heterotrimer Vps26-Vps29-Vps35 and members of the sorting nexins, orchestrates several cargo-sorting activities, including retrograde trafficking between endosomes and the TGN and endosome-to-plasma membrane recycling transport [51, 52]. One notable cargo that depends on retromer for its retrieval to the Golgi is the Wnt-sorting receptor Wntless/MIG-14 [51, 52]. Several studies have demonstrated that mutations in *Drosophila Vps35* impair Wnt secretion by affecting the recycling of its sorting receptor Wntless [53–55]. Based on the above data, and the known role of Wnt signaling in cancer [56], it has been proposed that GOLPH3 and the retromer might regulate the endosomes-to-Golgi transport of Wnt and other membrane receptors, determining effects on signaling pathways that are relevant for cancer disease [56]. Consistent with this hypothesis, loss of Vps35 disrupts endocytosis in *Drosophila* blood cells resulting in enhanced levels of several surface receptors at the plasma membrane including the EGF receptor and the platelet-derived growth factor [57].

### Golph3 and glycosyltransferases

Recent data have also suggested that GOLPH3 proteins might regulate the process of protein glycosylation in the Golgi [58]. Glycosylation, the enzymatic addition of monosaccharides or polysaccharides (glycans) to proteins or lipids plays crucial roles in the metabolism of eukaryotic cells [59–61]. Synthesis of N-linked or O-linked glycans starts in the endoplasmic reticulum (ER), is completed in the Golgi and requires the sequential action of several glycosyltransferases, glycosidases and nucleotide-sugar transporters [59]. GOLPH3 family proteins have been involved in proper docking and localization of Golgi-resident glycosyltransferases [58]. Golgi glycosyltransferases are all type II integral membrane proteins consisting of a short cytoplasmically exposed N-terminal region, a single membrane-spanning segment and a luminal enzymatic domain [59]. Coatomer (coat protein COP I) coated vesicles represent the predominant means for retrograde traffic of Golgi enzymes within the Golgi [62]. However the cytoplasmic tails of Golgi glycosyltransferases lack COP I sorting motifs [40]. Yeast Vps74p is required to maintain the steady-state localization of Golgi glycosyltransferases by binding the cytosolic portions of these enzymes [40]. Tetramerization of Vps74p is crucial for binding of this protein to a pentameric sequence motif at the cytoplasmic tail of a subset of Golgi-glycosyltransferases [40]. Vps74p also binds directly to coatomer, the vesicle coat complex that mediates retrograde trafficking [40]. Based on these results, Tu and coauthors [40] proposed that Vps74p might contribute to maintain the steady-state localization of these Golgi-resident enzymes by acting as a sorting receptor for the COPI coat. Vps74p and GOLPH3 proteins contain a cluster of arginine residues at the N-terminal that is necessary and sufficient for binding to coatomer (Figure 1B) [63].

The consensus amino acid sequence (F/L)(L/I/V)*XX*(R/K), that is recognized by Vps74p in the cytoplasmic tails of yeast glycosyltransferases, is also present in Core 2N-acetylglucosaminyltransferase 1 (C2GnT1) of several other species including *Homo sapiens* [64]. Human C2GnT1 is an important enzyme during the synthesis of core 2-associated sialyl Lewis^x^ (C2-O-sLex), a selectin ligand involved in leukocyte trafficking and metastatic tumors [64]. Human GOLPH3 interacts with C2GnT1 in the myeloid cell line KG1a and mediates its Golgi localization by binding to the cytoplasmic tail of this protein [64]. However recent studies reported that GOLPH3 protein binds to certain Golgi glycosyltransferases even when they lack the pentameric motif in their cytoplasmic tails [65–67]. The mammalian gene *POMGnT1* encodes the O-mannosyl-β-1,2-N-acetylglucosaminyltransferase 1, a glycosyltransferase that catalyzes the second step in the O-mannosylation of α-dystroglycan [65]. The α-dystroglycan glycoprotein is a key component of the α-dystroglycan complex that mediates interaction with laminin and other components of the extracellular matrix [68]. Alterations of the glycosylation status of α-dystroglycan disrupt the interaction between this protein and the extracellular matrix causing various forms of congenital muscular dystrophies [69, 70]. The interaction of POMGnT1 cytoplasmic tail and GOLPH3 is essential to mediate localization of this glycosyltransferase to the Golgi [65]. RNAi mediated knockdown of GOLPH3 impairs retaining of POMGnT1 at the Golgi and determines a reduction in IIH6 immunoreactivity indicating a defective glycosylation status of α-dystroglycan [65].

The *Drosophila* GOLPH3 was shown to regulate the dynamic retrograde trafficking of exostosin-1 and exostosin-2, which are also involved in protein O-glycosylation [66]. These Golgi enzymes are type II transmembrane glycosyltransferases that add D-glucuronic and N-acetylglucosamine units in the biosynthesis of heparane sulfate proteoglycans [66]. In human, mutations in the exostosin genes *EX1* and *EX2* disrupt the synthesis of glycosaminoglycan and are associated with multiple osteochondrosomas characterized by the development of multiple cartilaginous tumors [71]. In *Drosophila,* both loss of function mutations and overexpression of GOLPH3 disrupt dynamic retention and protein stability of exostosins resulting in defective synthesis of heparin sulfate proteoglycans and reduction in Hedgehog signaling [66]. The requirement for GOLPH3 protein in N-glycosylation processes was also demonstrated [67]. Isaji and coauthors [67] showed that human GOLPH3 associates specifically with sialyltransferases and affects sialylation of N-glycans especially α2,6 sialylation. Increased α2,6-linked sialylation on β1 integrins has been linked to cancer progression via increased cell motility [72]. Strikingly Isaji and coauthors reported a positive correlation between the sialylation of β1 integrins end the expression levels of GOLPH3 [67]. Furthermore overexpression of α2,6-sialyltransferase-I (SiaT) rescues cell migration and signaling alterations caused by GOLPH3 depletion [67]. The involvement of human GOLPH3 in protein glycosylation was also demonstrated by work of Eckert and coauthors [73]. They reported the biochemical interaction of human GOLPH3 protein with C2GnT and SiaT but not with β1,4-galactosyltransferase I (GalT) [73]. Importantly, by using two different biochemical assays they found the presence of GOLPH3, C2GnT and SiaT in COPI–coated vesicles [73]. Moreover they demonstrated that GOLPH3 is not required for vesicle formation. However depletion of GOLPH3 affects proper incorporation of C2GnT and SiaT in COPI vesicles [73]. Conversely galactosyltransferase, which does not interact with GOLPH3, is not dependent on GOLPH3 for its localization [73].

Importantly altered glycosylation is a universal feature of malignant transformation and cancer progression [59]. Indeed several structural changes in cell surface N- and O-glycans have been observed in cancer cells including high mannose *N*-linked glycans, sialylation and fucosylation of N- and/or O-linked glycans, expression of short O-glycan residues (such as sT/Tn) and sialylated Lewis antigens [74]. Moreover there is accumulating evidence demonstrating that cancer-associated glycan structures, exposed on the cell surface, are associated with pathological processes linked to cancer disease such as the migratory, invasive and metastatic behaviors and the abnormal signal transduction pathways [74, 75]. Taken together these data suggest that the involvement of GOLPH3 in protein glycosylation represents a relevant aspect of its oncogenicity.

### GOLPH3 and mTOR signaling

In the context of cancer pathogenesis, GOLPH3 was shown to enhance signaling through the mammalian target of rapamycin (mTOR) [5]. The TOR kinase, which is the catalytic subunit of the TORC1 and TORC2 signaling complexes, is crucial to regulate cell growth, cell proliferation and survival in eukaryotic cells [76]. GOLPH3 overexpression enhances the activity of both mammalian TORC1 and TORC2 complexes resulting in elevated phosphorylation of their respective substrates S6 Kinase and AKT [5]. Human melanoma cells overexpressing GOLPH3 develop tumors faster than control cells when transplanted into immunodeficient animals [5]. Moreover GOLPH3-expressing tumors are also much more sensitive to rapamycin indicating that the oncogenic activity of GOLPH3 is mediated through mTOR signaling [5]. A link between TOR and the retromer trafficking was supported by work in budding yeast [77]. Aronova and coauthors [77] showed that Tor1p and Torp2 (the orthologs of mTOR1 and mTOR2) fractionate with special detergent-resistant membranes that are enriched for proteins involved in either actin cytoskeleton organization or endocytosis. Furthermore the authors demonstrated that TOR1/2 proteins associate with endocytic markers and that inhibition of TORC1 by rapamycin impairs fluid-phase endocytosis. They also found a considerable number of genetic interactions between components of yeast TORC1 and actin/endocytosis related genes. By combining the results from genetic analysis with database mining they could define a network of functional interactions between TORC1 and vesicle trafficking components. Remarkably two genes of this network encode the Vps35p and Vps29p subunits of the retromer complex. These data revealed a link between TOR complexes and the retromer-endocytic trafficking. Given the interaction between GOLPH3 and the retromer component Vps35, it has been proposed that upregulation of GOLPH3 might affect mTOR complexes by promoting recycling of key membrane receptors [58]. Alternatively, the proved association of GOLPH3 with glycosyltransferases suggests that deregulation of this protein might impinge upon mTOR signaling by affecting the glycosylation status or the secretion of a relevant glycoprotein connected with this pathway [58].

### Golph3 regulates cell migration and invasion

Recent studies have implicated GOLPH3 in cell migration and invasion in glioma and breast cancer cells [8, 11, 12]. In glioma cells the molecular mechanism underlying GOLPH3 function in migration and invasion requires mTOR and one of its effectors, the transcription/translation regulatory protein Y-box binding protein-1 (YB1) [12]. Previous work demonstrated that YB-1 levels are increased in invasive breast cancer cells and linked with reduced expression of E-cadherin [78]. Moreover in noninvasive breast epithelial cells, YB-1 was shown to promote the epithelial-mesenchymal transition (EMT), a process by which epithelial cells lose apical-basal polarity and cell-cell contact to acquire a motile, mesenchymal-like phenotype [78]. The recent study of Zhang and coauthors [12] demonstrated a direct correlation between GOLPH3, YB-1 and mTOR in glioma cancer cells such that GOLPH3 up-regulation is accompanied by an increase of YB1 level and mTOR activity. Furthermore GOLPH3 promotes the migratory and invasive behavior of glioma cells *in vitro* and this effect is abolished by either treatment with the mTOR inhibitor INK128 or by YB1 knockdown [12].

Tokuda and coauthors [8] reported that high levels of Golgi PI(4)P enhance cell migration/invasion capacity of breast cancer cells through a mechanism that depends on GOLPH3 and its ability to interact with PI(4)P. In the triple-negative breast cancer cell line, MDA-MB-231 cells, cell-cell adhesion is impaired and cell migration is promoted by overexpression of wild type GOLPH3 but not by the R90L mutant version of this protein. Moreover the enhanced invasion ability observed in GOLPH3 expressing cells is abolished by silencing the PI4KIIIβ that generates PI(4)P in the Golgi [8]. As previously said, GOLPH3 function has been also associated with integrin-mediated cell migration through its role in promoting sialylation of integrin N-glycans [67].

### GOLPH3 and mitochondrial functions

GOLPH3 was also identified during a screen for proteins whose expression is increased after depletion of mitochondrial DNA (mtDNA) [33]. Following an immunostaining procedure that entails permeabilization pretreatment, Nakashima-Kamimura and coauthors [33] reported that, besides localizing to the Golgi, GOLPH3 protein was also enriched to mitochondria. These researchers also found that expression of GOLPH3 is enhanced, as a consequence of mitochondrial dysfunction, in muscle fibers of patients suffering from the mitochondrial diseases CPEO (chronic progressive external ophthalmopelia) and MELAS (mitochondrial myopathy, encephalopathy, lactic acidosis and stroke-like episodes) [33]. In addition in HeLa cells that stably express GOLPH3, the levels of mitochondrial phospholipid cardiolipin increase by 1,75 fold coupled with an increase of total mass of mitochondria revealed by three-dimensional imaging. Based on these results it was proposed that GOLPH3 might control mitochondrial mass through a mechanism that depends upon mitochondrial lipids [33]. By shuttling between the Golgi apparatus and mitochondria this protein might regulate the delivery of cardiolipin and other mitochondrial lipids and thereby the overall mitochondrial mass [33].

Recent studies indicate that mitochondria play a fundamental role for cancer metabolism [79]. Several data have suggested that human tumors consist of two co-existing metabolic compartments [34, 79, 80]. It was shown that the enhancement of mitochondrial oxidative metabolism in breast cancer cells leads to the release of H_2_O_2_ and reactive oxygen species into the tumor microenvironment [34, 79, 80]. This in turn results in loss of Caveolin-1 protein and induction of autophagy and mitophagy in the stromal fibroblasts with a consequent reduction of the number of mitochondria that shifts the metabolism of these cells towards aerobic glycolysis [80]. The enhanced glycolysis in cancer-associated fibroblasts generates excessive lactate, pyruvate and ketone bodies, which are secreted into the intracellular space. These metabolites are then used by neighboring cancer cells for their mitochondrial oxidative metabolism, resulting in an increase of mitochondrial mass [35]. Remarkably Salem and coauthors [35] demonstrated that overexpression of GOLPH3 in MDA-MB-231 triple-negative breast cancer cells promotes mitochondrial biogenesis as revealed by a 2–3-fold increase in Mito Tracker activity. In addition these researchers observe a 3-fold increase in tumor growth (relative to the vector alone control) when cancer cells overexpressing GOLPH3 are injected into the flanks of athymic nude mice [35]. Importantly when hTERT fibroblasts overexpressing GOLPH3 are co-injected with MDA-MB breast cancer cells, they fail to affect tumor growth indicating that the effects of GOLPH3 on tumorigenesis depend on the specific tumor compartment [35].

### Golph3 is required for cleavage furrow formation during cytokinesis

In animal cells cytokinesis accomplished by the constriction of a contractile ring [81]. This cytoskeletal structure, composed of actin filaments and Myosin II, is tightly anchored to the plasma membrane of the dividing cell through a network of scaffolding proteins including Septins and Anillin [82, 83]. The spatial information that directs contractile ring assembly and Myosin activation depends on the central spindle microtubules that deliver key regulators of the Rho GTPase at cell equator [84, 85]. Successful cytokinesis also requires membrane trafficking, from both the secretory/recycling pathways, to the cleavage furrow [86, 87]. Evidence indicated that contractile ring assembly and dynamics as well as new membrane addition depend on special lipids at the cleavage site [88, 89]. Polyanionic lipids, particularly Phosphatidylinositol 4,5-biphosphate and its precursor PI(4)P, fulfill important functions for cleavage furrow ingression [88, 89]. Work in Dr. Brill lab involved PI(4)P in *Drosophila* cytokinesis [90]. Polevoy and coauthors showed that the PI4KIIIβ Fwd directly binds the small GTPase Rab11 and is required for synthesis of PI(4)P on Golgi membranes [91]. Although Fwd protein does not concentrate at the cleavage furrow, its function is essential for localization of secretory organelles containing both PI(4)P and Rab11 at the cleavage site of male meiotic cells [91]. The phosphoinositide PI(4,5)P2 was visualized at plasma membrane of the cleavage furrow in dividing tissue culture cells and *Drosophila* male meiotic cells [89, 92, 93]. Polyanionic lipids were also shown to affect the activity of several components of the cytokinesis machinery. PI(4,5)P2 interacts *in vitro* with Septins and modulates F-actin polymerization by regulating the activity of the actin binding proteins profilin and cofilin [94, 95]. In addition several components of the Rho signaling pathway including Rho A, the Rho GEF ECT2 and the centralspindlin subunit MgcRacGAP, contain protein domains that can mediate the interaction with PI(4,5)P2 or PI(4)P [96–98].

Our recent work demonstrated the requirement for GOLPH3 in cytokinesis [29]. *Drosophila* GOLPH3 protein is essential for normal cytokinesis and accumulates at the cleavage site of dividing spermatocytes and larval neuroblasts. Our data indicated that GOLPH3 function in cytokinesis is strictly dependent on its ability of this protein to bind PI(4)P. Mutations in GOLPH3 that abolish interaction with PI(4)P (Figure 3), impair its localization to both the Golgi and the cleavage furrow. Moreover GOLPH3 protein forms a complex with Rab11 and is required to target both Rab11 and PI(4)P to the furrow site. Indeed both PI(4)P and Rab11 fail to accumulate at the cleavage site in spermatocytes from *GOLPH3* mutants. Visualization of PI(4,5)P2 by the PLCδ-PH-GFP fusion protein, reveals enrichment of PI(4,5)P2 at the cleavage furrow in wild type but not in *GOLPH3* mutants. Remarkably our work suggested a possible interaction of GOLPH3 with phosphatidic acid (PA), a glycerophospholipid that modulates plasma membrane curvature and activates PI(4)P 5-kinase [99, 100]. Thus, through binding to PA, GOLPH3 might be implicated in PI(4)P 5-kinase activation and plasma membrane deformation during furrow ingression (Figure 3). Biochemical analyses also indicate interactions of GOLPH3 protein with components of the cytokinesis machinery. Consistent with these data, wild type function of GOLPH3 enables maintenance of centralspindlin and Rho1 at cell equator and stabilization of Myosin II and Septin rings. Based on these results we have proposed that GOLPH3 plays a key role in coupling phosphoinositide signaling with actomyosin dynamics during cytokinesis (Figure 3). The presence of cytokinesis failures and tetraploid metaphases in somatic cells of *Drosophila* larval brains that were depleted of GOLPH3, demonstrates the requirement for this protein for normal cytokinesis after mitosis [29]. Since tetraploidy has been associated with cancer initiation and progression [101–104] these findings suggest novel paths that can link the oncogene GOLPH3 to malignancy.

### Future perspectives and conclusions

Since the first demonstration of GOLPH3 oncogenicity, a large number of studies supported the correlation between GOLPH3 up-regulation and poor survival in many cancers, despite the anticancer chemotherapy used in oncology. These data have suggested that GOLPH3 can be an important biomarker of tumor progression [5–26]. It has also been reported that high levels of GOLPH3 protein enhance the frequency of cell death in rapamycin-treated melanoma cells, suggesting that GOLPH3 expression can be used to predict rapamycin sensitivity in tumor therapy [5]. However the anticancer efficacy of rapamycin and its analogs (rapalogs), which preferentially inhibit mTORC1 with a weak activity against mTORC2, has resulted modest at best [105].

Clinical cancer therapeutics is largely based on the use of DNA damaging agents [106, 107]; these include DNA alkylators such as cyclophosphamide, nitrosoureas and triazenas (e.g. dacarbazine and temozolomide), platinum compounds (e.g. cisplatin, carboplatin and oxaliplatin), nucleobase analogs (e.g. 5-fluorouracil), antifolates and topoisomerase poisons (e.g. camptothecin, the anthracyclines doxorubicin and daunorubicin). In this context, it's worth mentioning that overexpression of GOLPH3 enhances cell survival upon treatment with the DNA-damaging compounds camptothecin or doxorubicin suggesting that it can be used to evaluate the response to chemotherapy [36]. In addition Field and colleagues reported that knockdown of either GOLPH3, MYO18A or DNA-PK affects cell survival after DNA-damage indicating that the GOLPH3/MYO18A/DNA-PK pathway might be a potential effective therapeutic target [36]. Based on these findings, one therapeutic strategy might be to use small drugs that inactivate components of the GOLPH3/MYO18A/DNA-PK pathway in combination with traditional chemotherapeutic treatments based on DNA-damaging compounds. Given the striking evolutionary conservation of the GOLPH3 protein (Figure 1), simple eukaryotic model systems such as the fruit fly *Drosophila melanogaster* or the worm *Caenorhabditis elegans* might be useful in large-scale screening of small molecules that affect GOLPH3 activity.

In conclusion, although GOLPH3 protein has been described as a “first-in-class Golgi oncoprotein”, its cellular functions are not limited to maintenance of Golgi architecture and vesicle trafficking (Figures 2, 3). Indeed GOLPH3 protein has been involved in several processes that, if altered, can contribute to cell transformation and cancer progression, including mitochondrial function [33–35], cell adhesion [8, 11, 12], cellular response to DNA damage [36] and cytokinesis [29]. Given the strong connection between GOLPH3 expression and cancer survival, a comprehensive knowledge of the molecular circuits requiring this protein will be essential to identify novel cancer therapeutic strategies.
